# Pseudarthrosis risk factors in lumbar fusion: a systematic review and meta-analysis

**DOI:** 10.1186/s12891-024-07531-w

**Published:** 2024-06-03

**Authors:** Win Boonsirikamchai, Sirichai Wilartratsami, Monchai Ruangchainikom, Ekkapoj Korwutthikulrangsri, Sasima Tongsai, Panya Luksanapruksa

**Affiliations:** 1 Department of Orthopaedics Surgery, Faculty of Medicine, Siriraj Hospital, Mahidol University, Wang Lang Rd., Bangkok, 10700 Thailand; 2 Office for Research and Development, Faculty of Medicine, Siriraj Hospital, Mahidol University, Wang Lang Rd., Bangkok, 10700 Thailand; 3 Department of Orthopaedics, Bhumibol Adulyadej Hospital, Phahon Yothin Rd., Bangkok, 10220 Thailand

**Keywords:** Pseudarthrosis, Lumbar vertebrae, Lumbar fusion, Spinal fusion, Risk factors

## Abstract

This study presents a systematic literature review and meta-analysis of pseudarthrosis risk factors following lumbar fusion procedures. The odds ratio (OR) and 95% confidence interval (95% CI) were used for outcome measurements. The objective of this study was to identify the independent risk factors for pseudarthrosis after lumbar spinal fusion, which is crucial for mitigating morbidity and reoperation. Systematic searches in PubMed, Embase, and Scopus (1990–July 2021) were conducted using specific terms. The inclusion criteria included prospective and retrospective cohorts and case‒control series reporting ORs with 95% CIs from multivariate analysis. The quality assessment utilized the Newcastle–Ottawa scale. Meta-analysis, employing OR and 95% CI, assessed pseudarthrosis risk factors in lumbar fusion surgery, depicted in a forest plot. Of the 568 abstracts identified, 12 met the inclusion criteria (9 retrospective, 2006–2021). The 17 risk factors were categorized into clinical, radiographic, surgical, and bone turnover marker factors. The meta-analysis highlighted two significant clinical risk factors: age (95% CI 1.02–1.11; *p* = 0.005) and smoking (95% CI 1.68–5.44; *p* = 0.0002). The sole significant surgical risk factor was the number of fused levels (pooled OR 1.35; 95% CI 1.17–1.55; *p* < 0.0001). This study identified 17 risk factors for pseudarthrosis after lumbar fusion surgery, emphasizing age, smoking status, and the number of fusion levels. Prospective studies are warranted to explore additional risk factors and assess the impact of surgery and graft type.

## Introduction

The introduction elucidates the consequentiality of symptomatic pseudarthrosis subsequent to a spinal fusion procedure, complementing its suggestions for postoperative morbidity and the imperative for reoperation. The pivotal role of fusion status after posterolateral lumbar fusion (PLF) in dictating long-term outcomes for lumbar canal stenosis treatment is underscored [1]. The acknowledged financial and quality-of-life ramifications of pseudarthrosis underscore the urgency of addressing this complication [2].

The literature cited in the introduction serves as the underpinning for the study’s rationale. The documented incidence of pseudarthrosis post lumbar fusion surgery (5–15%) [3] and its substantial contribution to revision fusion surgery (23.6%) [4] underscore the clinical pertinence of this complication. Nunna et al.’s revelations regarding smoking as a global risk factor for pseudarthrosis [5], coupled with Glassman et al.’s identification of a significant dichotomy in pseudarthrosis rates between smokers and nonsmokers, contributed to a nuanced understanding of the multifaceted nature of the condition [6].

The introduction also references Raizman et al.’s delineation of pivotal factors influencing fusion rates in lumbar spine surgery, encompassing instrumentation type, fusion location, graft type, and brace type [4].

Lee et al.’s study added the intricacy of risk factors by spotlighting fusion length and the adipose content of paraspinal muscle as determinants influencing union rates [7]. Gologorsky et al.’s revelations concerning, and construct type as linked to pseudarthrosis further underscore the diversity of risk factors [8].

Other studies identifying age, DM (presumably diabetes mellitus), BMI, and cage subsidence as noteworthy risk factors for pseudarthrosis have broadened the spectrum of potential contributory factors. Through the amalgamation of these findings, the introduction underscores the intricate and multifaceted nature of pseudarthrosis risk factors.

The significant reasoning for the review radiates from the lacunae in the ongoing review. The challenge of pre-emptively predicting pseudarthrosis has been underscored, and the imperative for advancing both short-term and long-term patient outcomes through the discernment of risk factors has been accentuated. Preventive measures provided a pragmatic impetus for this research, suggesting that an enhanced understanding and targeted addressing of these risk factors could lead to a decrease in the incidence of pseudarthrosis subsequent to lumbar spinal fusion procedures.

In summary, the introduction establishes the clinical gravity of pseudarthrosis, articulates existing knowledge on its risk factors from diverse studies, and feature the exigency for an extensive survey to blend and enhance understanding in this complicated field.

## Methods

This systematic review was synthesized according to the Preferred Reporting Items for Systematic Reviews and Meta-Analysis (PRISMA) protocol (2020).

### Data source and search strategy

We searched PubMed, Embase, and Scopus for all studies from 1990 to July 2021 reporting pseudarthrosis risk factors after lumbar fusion surgery. The following search terms were used: (“risk factor” or “factors”) AND (“pseudarthrosis” or “nonunion”) AND (“lumbar fusion” or “lumbar arthrodesis” or “lumbar interbody fusion”). The search limits were the English language, studies were conducted on humans, and the full text was available. The inclusion criteria were prospective, retrospective cohort, and case‒control studies that reported risk factors for pseudarthrosis, odds ratios (ORs), and 95% confidence intervals (95% CIs) in patients who underwent lumbar fusion surgery. The exclusion criteria were no risk factors reported, no multivariate analysis, no odds ratio or 95% confidence interval reported, other sites of spinal fusion, fewer than 30 patients, tumor or neuromuscular disorders, other types of publication, patients from insurance databases, and unavailable full text. Additional articles relevant to risk factors for pseudarthrosis were identified from the reference lists of the retrieved studies. Both reviewers (WB, PL) independently screened abstracts and titles after removing duplicated publications. Afterward, full paper readings were performed to determine final inclusion. A study that reported risk factors from the multivariable analysis without 95% CIs was included in the qualitative analysis but excluded from the quantitative analysis. Disagreements were resolved by discussion to reach a consensus.

### Quality assessment

Two reviewers (WB, PL) independently performed the quality assessment of the included studies using the Newcastle–Ottawa Scale for cohort and case–control studies, with total score ranges of 0–9 calculated from three major categories, namely, selection, comparability, and outcome [9].

### Data extraction and outcome measurement

This review focused on the risk factors for pseudarthrosis after lumbar fusion procedures. Two reviewers (WB, PL) independently extracted the following data from the multivariable analysis: the first author, name of the journal, study design, year of publication, year of data collection, number of patients, mean or median age of the sample, diagnosis, fusion procedure, graft types, time of final follow-up, pseudarthrosis criteria, independent pseudarthrosis risk factors, odds ratio (OR) and 95% confidence intervals (CIs). Disagreements were discussed until consensus was achieved.

### Data analysis and statistical analysis

Risk factors affect the incidence of pseudarthrosis in patients who underwent lumbar fusion surgery. The odds ratio was used as the primary effect estimate in the meta-analysis. Only the odds ratios (ORs) and 95% confidence intervals (CIs) of the variables reported as significant predictors in at least two papers were pooled in the meta-analysis. Statistical heterogeneity was assessed using the Cochrane Q test, with a *p* value set at 0.1 indicating statistical significance. Heterogeneity between studies was evaluated based on the inconsistency (I^2^) index, and sub substantial heterogeneity was represented by an I^2^ > 50%. The common effect model was used when the effects were assumed to be homogeneous. In the presence of heterogeneity, we used a random effects model. Sensitivity analysis was performed by omitting studies one at a time to investigate the effect on the overall meta-analysis result. We rejected the studies that caused greater statistical heterogeneity (I2 > 90). A *p* value < 0.05 was considered to indicate statistical significance. Publication bias was assessed using a funnel plot, Begg test, and Egger test. A trim-and-fill method was used to estimate the pooled odds ratio after adjusting for funnel plot asymmetry arising from publication bias. The meta-analysis was performed using the meta package (R Development Core Team, 2015, Vienna, Austria) version 3.2.2.

## Results

### Included studies

**Fig. 1:**
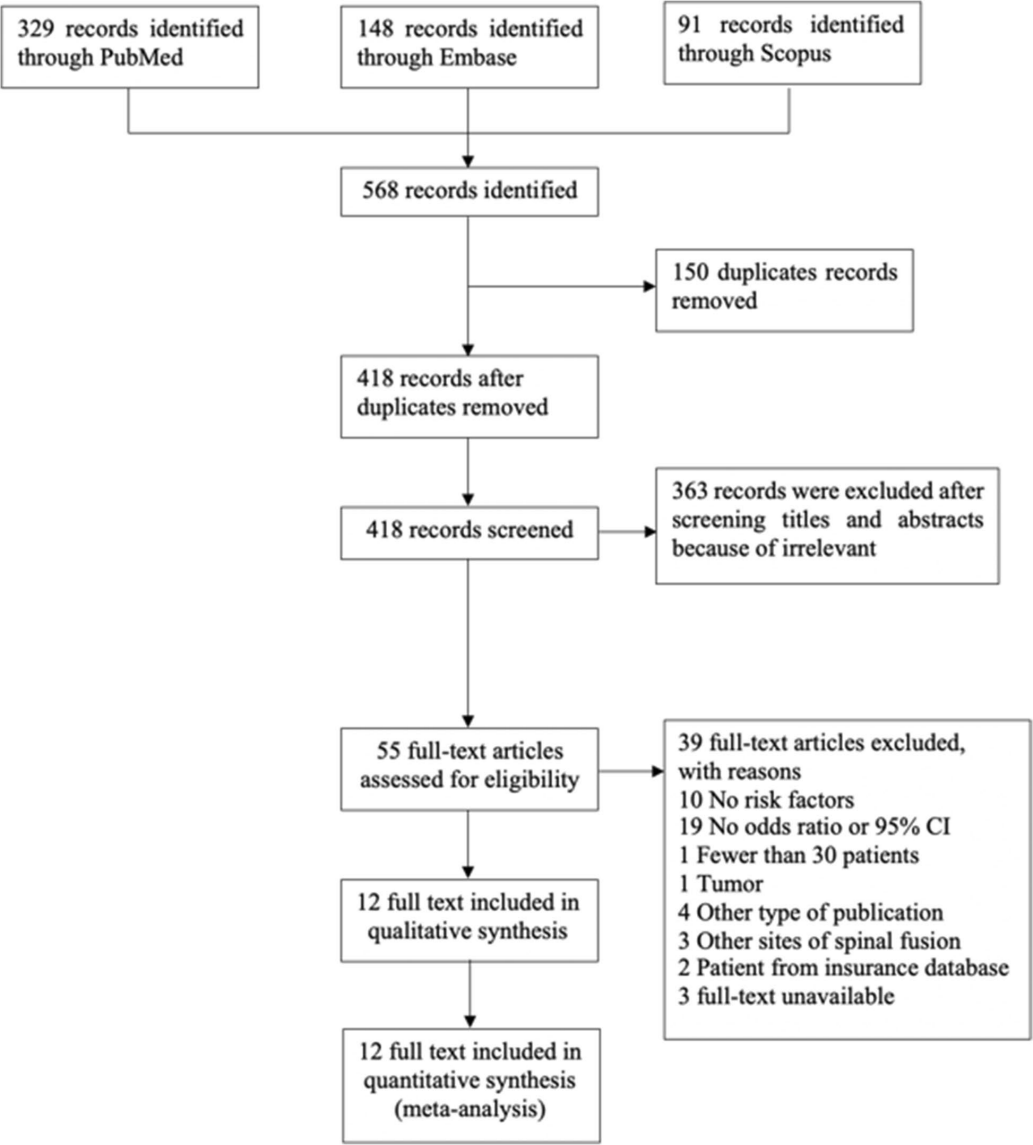
The PRIASMA flow diagram illustrates the studies that have been identified, included and excluded as well as the reason for exclusion

A total of 568 abstracts (329 from PubMed, 148 from Embase, and 91 from Scopus) were identified through a database search. There were 150 duplicate publications; thus, 418 unique abstracts were screened. Among these abstracts, 55 were selected for full-text review, and 39 articles were excluded for the following reasons: 10 had no risk factors for pseudarthrosis reported, 19 did not report odds ratios or 95% CIs, 1 had fewer than 30 patients, 1 had a diagnosed tumour, 4 had other types of publication (review article, case report, or case series), 3 had other sites of spinal fusion, 2 had patients from the insurance database and 3 had unavailable full-text data. Finally, the reviewers selected a total of 12 studies for systematic review and meta-analysis. A flow diagram of the literature search is shown in Fig. 1.

### Characteristics and quality of the included studies

A total of 1,830 patients were enrolled in 12 included studies. The vast majority of the studies were retrospective (9 studies) and were published between 2006 and 2021. Two studies were prospective cohort studies [10, 11]. One was a case‒control study [12]. The number of enrolled patients ranged from 63 to 416. The age of the enrolled patients ranged from 53.9 ± 9.6 to 72.1 ± 6.9 years. The most common diagnosis was spinal stenosis [13–19]. Among the surgical types, five studies performed PLF [11, 13, 14, 16, 20], five studies performed transforaminal lumbar interbody fusion (TLIF) [12, 15, 16, 18, 21], two studies performed lateral lumbar interbody fusion (LLIF) [17, 19], one study performed posterior lumbar interbody fusion (PLIF) [18], and one study performed oblique lateral lumbar interbody fusion (OLIF) [21]. Five studies used local grafts [12–15, 18], four studies used iliac crest bone grafts (IBG) [11, 13–15], three studies used recombinant bone morphogenic protein-2 (RH-BMP2) [13, 14, 20], two studies used cancellous allografts [17, 19], and only one study used demineralized bone matrix (DBM) [21]. The follow-up time was 12 months in five studies [12–14, 16, 18], 24 months in seven studies [10, 15, 17–21], and 60 months in one study [11].

The most common pseudarthrosis criterion used was more than 3 degrees of motion on a flexion–extension radiograph [11–15, 17, 18] followed by an absence of bridging bone and radiolucent around screws on CT [12–15, 17, 18]; one study used grades 3–4 from the Modified Bridwell criteria [21]. The characteristics of the included studies are shown in Table 1.

**Table 1:** Characteristics of the included studies

Author	NOS	Publication year	Type	N	Mean age	Diagnosis	Surgery type	Fusion level	Graft type	F/U time(mo.)	Pseudarthrosis criteria	Risk factors	Odds ratio	95%CI
Bydon et al. [13]	7	2014	R	327	59.79	Spinal stenosis Spondylolisthesis	PLF	1–4	BMP Local IBG Allograft	12	absence of bridging bone in CT > 3^o^ motion on F/E film radiolucent bone around screw site in CT	Durotomy	2.23	1.05–4.75
Bydon et al. [14]	7	2014	R	141 140	58.91	Spinal stenosis Spondylolisthesis	PLF	2 1	BMP Local IBG Allograft	12	absence of bridging bone in CT > 3^o^ motion on F/E film radiolucent bone around screw site in CT	Smoking Smoking	3.97 0.84	1.26–12.51 0.17–4.13
Fujibayashi et al. [15]	7	2012	R	76	62.6	Spinal stenosis Spondylolisthesis	TLIF	-	Local IBG	25	absence of bridging bone in CT > 3^o^ motion on F/E film radiolucent bone around screw site in CT	Bone cyst Multioperation back Age Female Comorbid	166 12.4 1.1 2.4 3.1	22.4-inf 1.8–133 0.96–1.2 0.27–36.2 0.16–65.1
Hollern et al. [10]	6	2019	P	416	57.1	SSI following spinal fusion	All	-	All	24	-	Numbers of level fuse BMI	1.36 1.08	1.15–1.54 1.02–1.15
Inose et al. [16]	8	2017	R	74	70.4	Spinal stenosis	TLIF PLF	1–2	All	12	absence of bridging bone in CT radiolucent bone around screw site in CT	P1NP TRACP-5b Albumin	0.83 1.02 0.03	0.72-.09 1.006–1.03 0.001–0.38
Jung et al. [17]	7	2021	R	152	64.4	Spinal stenosis Spondylolisthesis	LLIF	1–4	Cancellous allograft	24	> 3^o^ motion on F/E film absence of bridging bone in CT	DM Smoking Fusion > 3 levels	2.82 6.50 2.53	1.31–6.08 1.68–25.17 1.09–5.87
Konomi et al. [18]	6	2020	R	78	66	Spinal stenosis Spondylolisthesis ASD	PLIF TLIF CBT-PLIF	1–2	Local	12	> 3^o^ motion on F/E film radiolucent bone around screw site in CT visible gap between endplate and cage on CT	Age > 75 Female J. surgeon Numbers of level fuse Bone cyst Cage subsidence Retropulsion CBT-PLIF	4.67 0.85 0.54 1.95 2.84 0.93 3.76 1.54	1.18–18.40 0.27–2.67 0.14–2.06 0.36–10.60 0.89–9.06 0.29–2.99 0.74–19.10 0.42–5.57
										24		Female Age > 75 J. surgeon Numbers of level fuse Bone cyst Cage subsidence Retropulsion CBT-PLIF	0.91 2.47 8.33 0.92 4.09 1.57 1.86 4.98	0.16–5.17 0.37–16.40 0.69–101 0.05–16.10 0.76–22.10 0.27–9.05 0.25–14.10 0.78–31.70
Lin et al. [21]	8	2019	R	67	67.9	Degenerative lumbar disease	OLIF TLIF	1–3	DBM HA	24	Grade 3–4 Modified Bridwell fusion criteria	Cage subsidence	17.24	1.67–178.09
Macki et al. [20]	7	2017	R	110	53.9	Degenerative lumbar disease	PLF	1–4	BMP	24	-	Smoking Age Numbers of level fuse	4.75 1.05 1.29	1.48–15.24 1.00–1.10 0.78–2.15
Otsuki et al. [12]	5	2021	CC	85	72.1	Degenerative lumbar disease	TLIF	1	Local	12	> 3^o^ motion on F/E film radiolucent bone around screw site in CT visible gap between endplate and cage on CT existence of an air intensity area inside interbody space	Age Mean of filling index Smoking BMD Cage type Mean of each maximum pedicle screw diameter	1.1 1.1 1.1 1 1.1 0.67	1.0–1.3 1.0–1.2 0.24–4.7 0.98–1.0 0.2–6.6 0.17–2.6
Satake et al. [19]	6	2018	R	63	69.8	Spinal stenosis Spondylolisthesis	LLIF	1–3	Cancellous allograft	24	no bone bridge formation connecting 2 vertebrae or between facing facet joint	Percutaneous pedicle screws usage BMI	3.14 0.88	1.13–8.68 0.76–1.01
Suda et al. [11]	7	2006	P	101	-	Isthmic spondylolisthesis	PLF	1–3	IBG	60	thin fusion mass > 3° motion on F/E film	Pre-op slip angle Pre-op %disc height	1.16 1.14	1.01–1.34 1.04–1.24

The median NRS score was 7. The score was seven in most of the studies [11, 13–15, 17, 20], eight in two studies [16, 21], six in three studies [10, 18, 19], and only one study [12]. The quality of the included studies measured by the NOS is shown in Table 2.

**Table 2:** NOS of the included studies

Author	Selection	Comparability	Outcome	NOS
Bydon et al. [13]	3	2	2	7
Bydon et al. [14]	4	1	2	7
Fujibayashi et al. [15]	3	1	3	7
Hollern et al. [10]	3	1	2	6
Inose et al. [16]	4	1	3	8
Jung et al. [17]	3	1	3	7
Konomi et al. [18]	4	1	1	6
Lin et al. [21]	4	1	3	8
Macki et al. [20]	3	2	2	7
Otsuki et al. [12]	2	1	2	5
Satake et al. [19]	3	1	2	6
Suda et al. [11]	4	1	2	7

### Risk factors for pseudarthrosis after lumbar fusion

A total of 17 risk factors for pseudarthrosis after lumbar fusion have been reported; these can be divided into clinical risk factors, radiographic risk factors, surgical risk factors, and bone turnover marker risk factors. The details of the risk factors are shown in Table 3.

**Table 3:** Risk factors of pseudarthrosis after lumbar fusion in categories

Risk categories	Risk factors
Clinical: -non modifiable	Age [12, 18, 20] Age > 75 [18] DM [17] Multioperation back [15] Smoking [13, 17, 20]
Clinical: modifiable	BMI [10]
Radiographic factors	Cage subsidence [21] Bone cyst [15] Preoperative disc height percentage [11] Preoperative slip angle [11] Mean of filling index [12]
Surgical factors	Number of levels fused [10] Fusion > 3 level [17] Percutaneous pedicle screw usage [19] Durotomy [14]
Bone turnover marker factor	Higher TRACP-5b [16] Lower P1NP [16]

Clinical risk factors can be divided into 2 groups: nonmodifiable and modifiable risk factors. The nonmodifiable risk factors included age [12, 18, 20], diabetes mellitus (DM) status [17], and multiple surgical procedures [15]. The modifiable risk factors included smoking [13, 17, 20] and BMI [10].

The radiographic risk factors for pseudarthrosis include cage subsidence [21], bone cyst [15], preoperative disc height percentage [11], preoperative slip angle [11], and mean filling index [12].

The surgical risk factors for pseudarthrosis followed by lumbar fusion include several levels of fusion [10], more than 3 levels of fusion [17], percutaneous pedicle screw usage [19], and durotomy [14].

From the systematic review, only one study by Inose et al. [16] revealed that a higher TRACP-5b concentration is a risk factor for pseudarthrosis in patients with lower P1NP levels. So, there is still no conclusion on turnover marker factors in meta-analysis.

The risk factors can be divided into two groups: the interbody fusion group and the posterolateral group (PLF). The details are shown in Table 4.

**Table 4:** Risk factor of pseudarthrosis divided in interbody fusion group and posterolateral fusion group

Categories	Interbody fusion	Posterolateral fusion (PLF)
Clinical: -non modifiable	Age [12] Age > 75 [18] DM [17] Smoking [17]	Smoking [13, 20]
Clinical: modifiable	Multioperation back [15]	
Radiographic factors	Cage subsidence [21] Bone cyst [15] Mean of filling index [12]	Preoperative disc height percentage [11] Preoperative slip angle [11]
Surgical factors	Number of levels fused [10] Fusion > 3 level [17] Percutaneous pedicle screw usage [19]	Durotomy [14]
Bone turnover marker factor	Higher TRACP-5b [16] Lower P1NP [16]	N/A

### Meta-analysis results

The 6 risk factors with similar variables that were mentioned in at least two studies were pooled in the meta-analysis. The details of the meta-analysis results are shown in Table 5.

**Table 5:** Show results of meta-analysis including pooled OR, 95% CI, sensitivity analysis, and publication

Risk factor	N	Total pts	Pooled OR	Pooled 95%CI	Heterogeneity (I^2^)	Model	*P*	Sensitivity analysis	Affected study	Begg	Egger
Age	3	271	1.06	1.02; 1.11	0.0%	Common	0.005	No effect	None	0.60	0.10
Female	2	154	1.26	0.31; 5.19	0.0%	Common	0.75	N/A	N/A	N/A	N/A
Number of level fuse	3	604	1.35	1.17; 1.55	0.0%	Common	< 0.0001	Effect	Hollern	0.12	0.007
Smoking	5	628	3.02	1.68; 5.44	36.2%	Common	0.0002	No effect	None	0.33	0.12
BMI	2	479	0.99	0.81; 1.21	85.6%	Random	0.90	N/A	N/A	N/A	N/A
Cage subsidence	2	145	4.57	0.44; 47.24	61.3%	Random	0.20	N/A	N/A	N/A	N/A

**Fig. 2:**
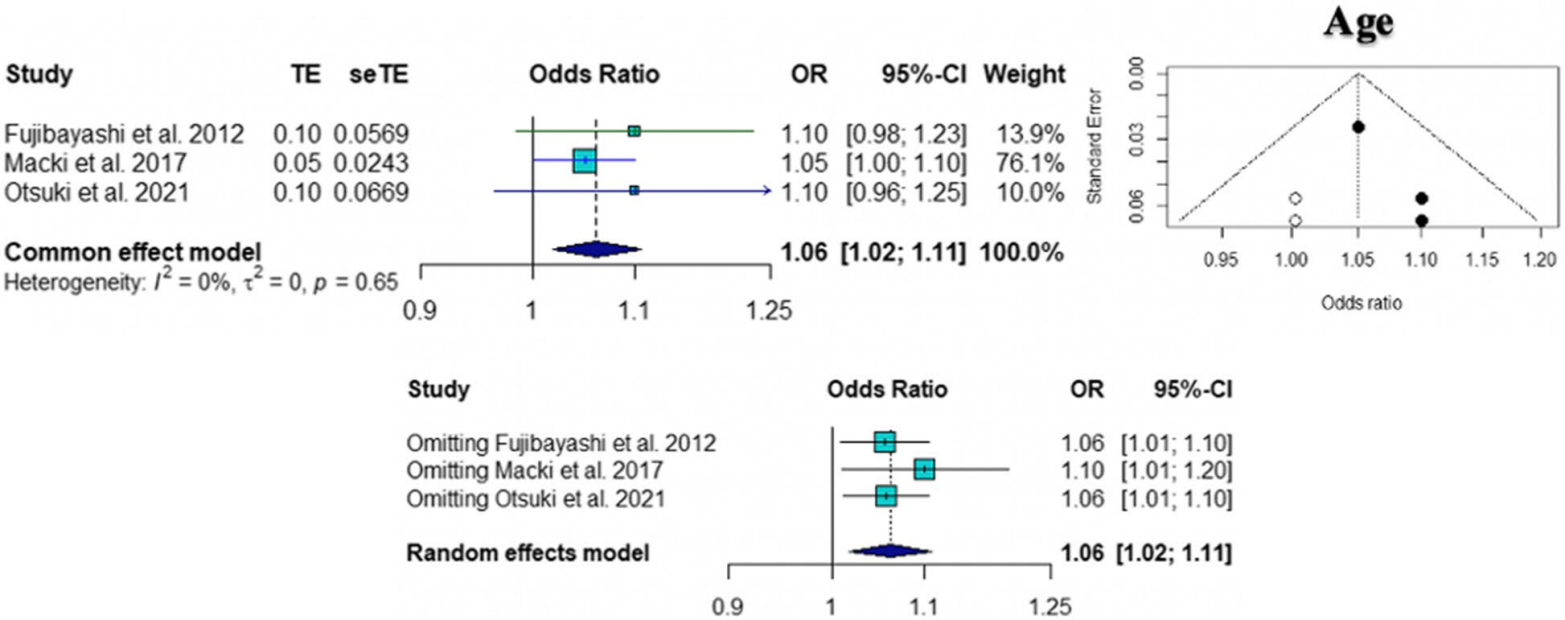
Forest plot, funnel plot and sensitivity analysis of pooled odds ratio for age

**Fig. 3:**
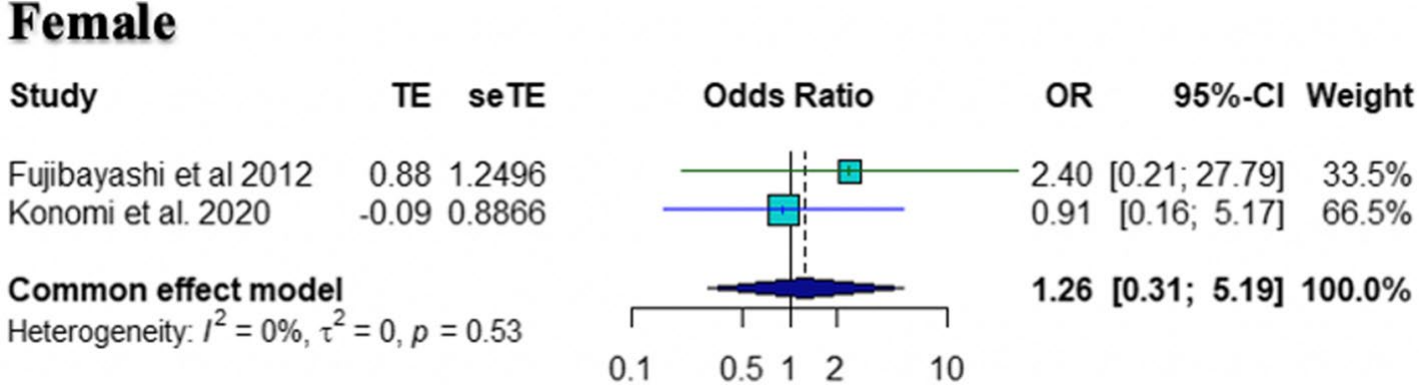
Forest plot showing pooled odds ratio and sensitivity analysis for female

**Fig. 4:**
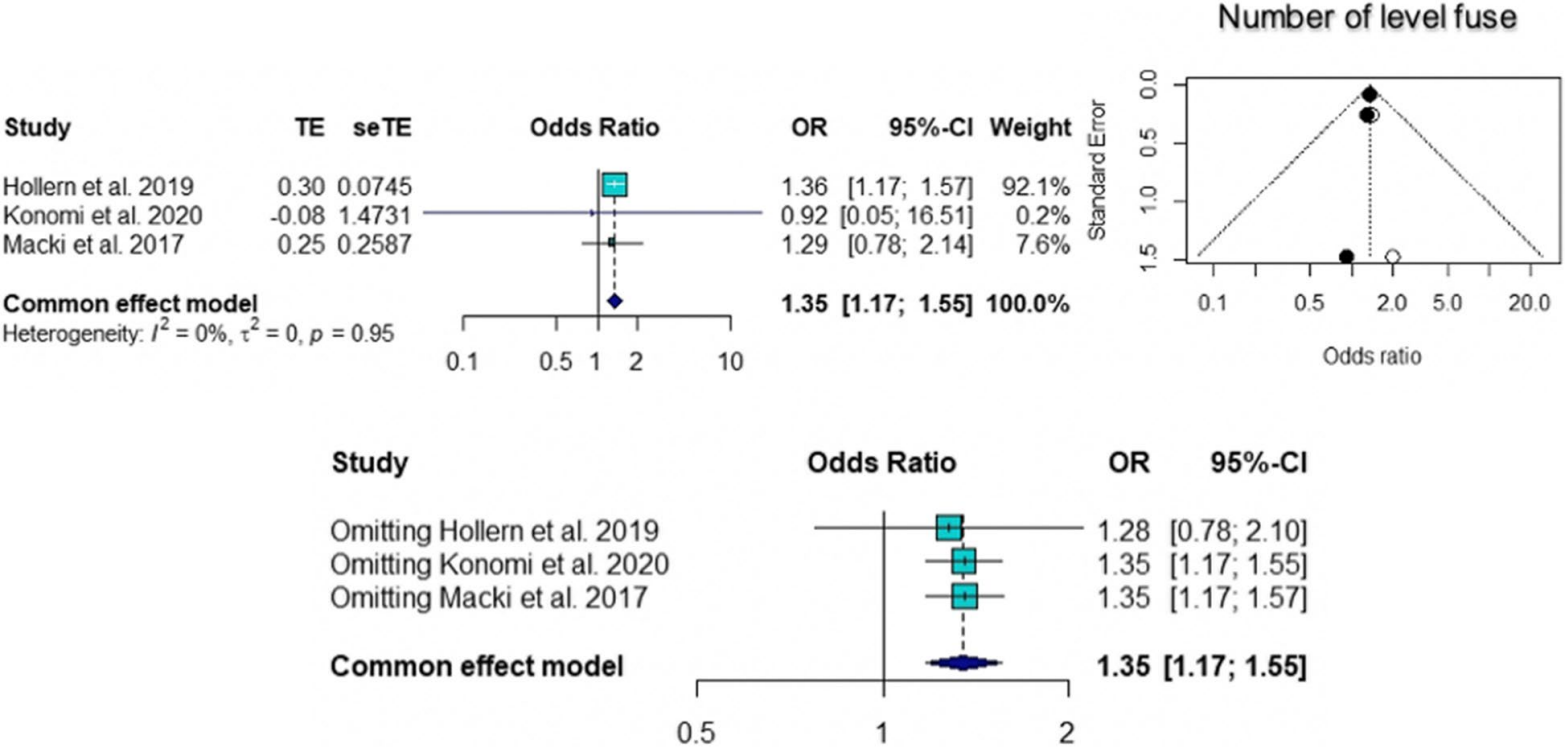
Forest plot, funnel plot and sensitivity analysis of pooled odds ratio for number of level fuse

**Fig. 5:**
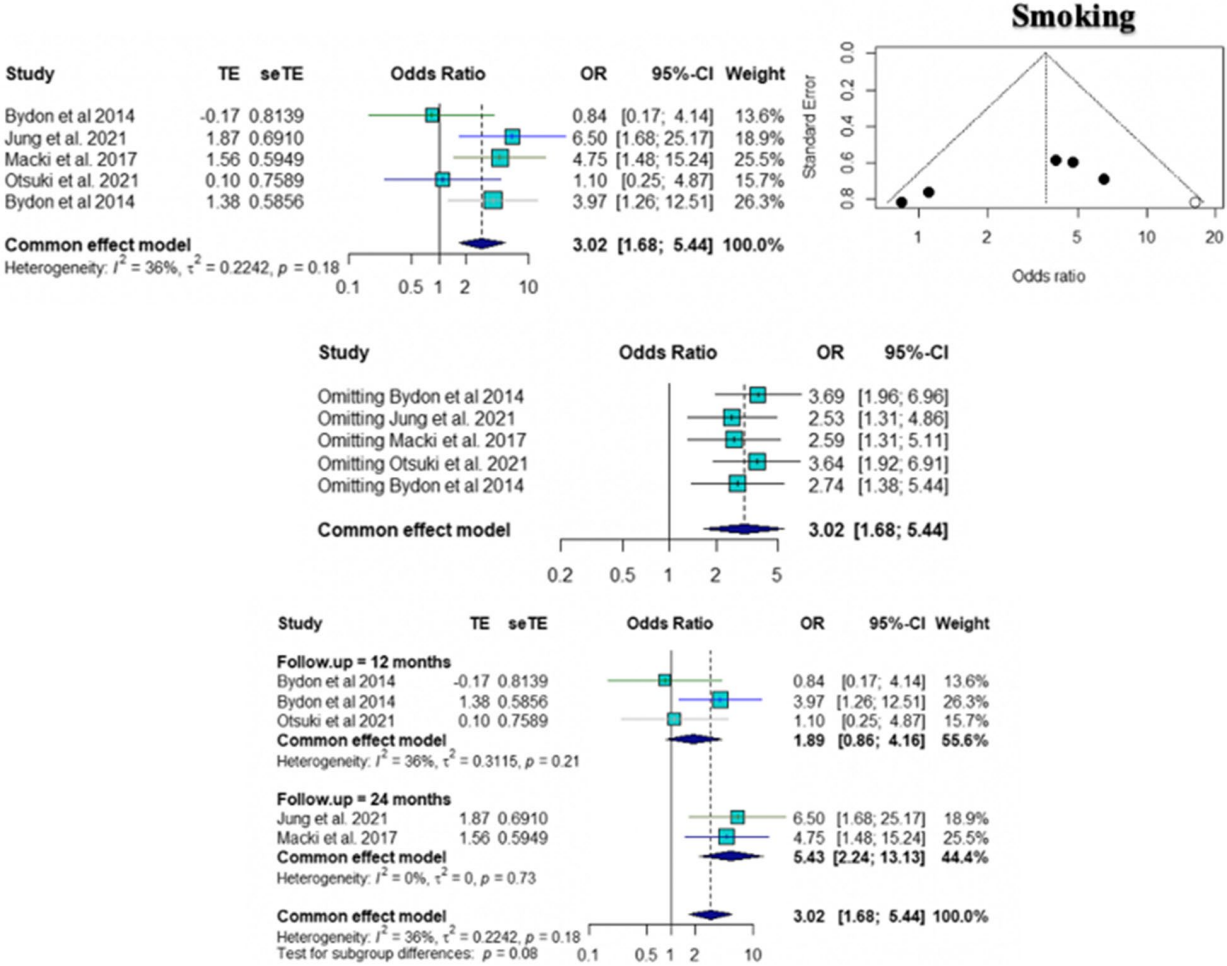
Forest plot showing pooled odds ratio and funnel plot showing publication bias for smoking, including forest plot of subgroup analysis according to time of follow up

**Fig. 6:**
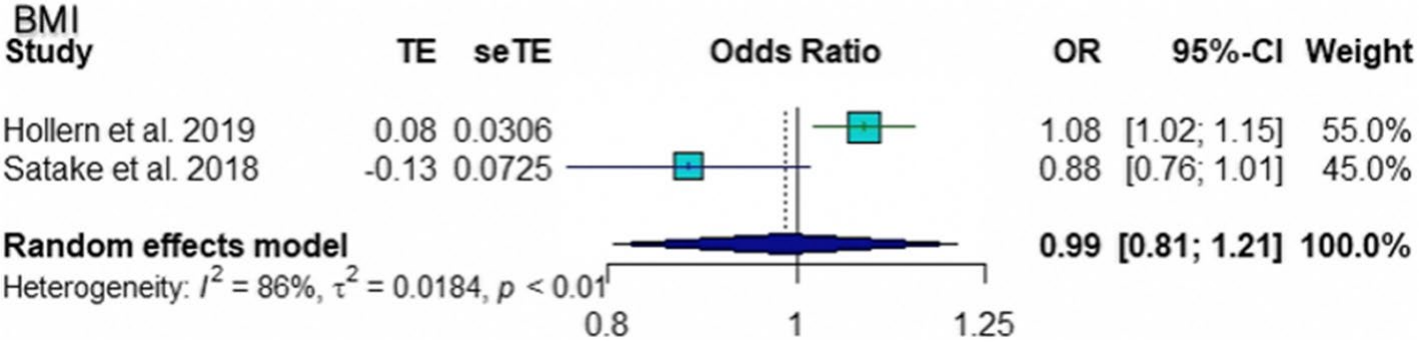
Forest plot and funnel plot of pooled odds ratio for BMI

**Fig. 7:**
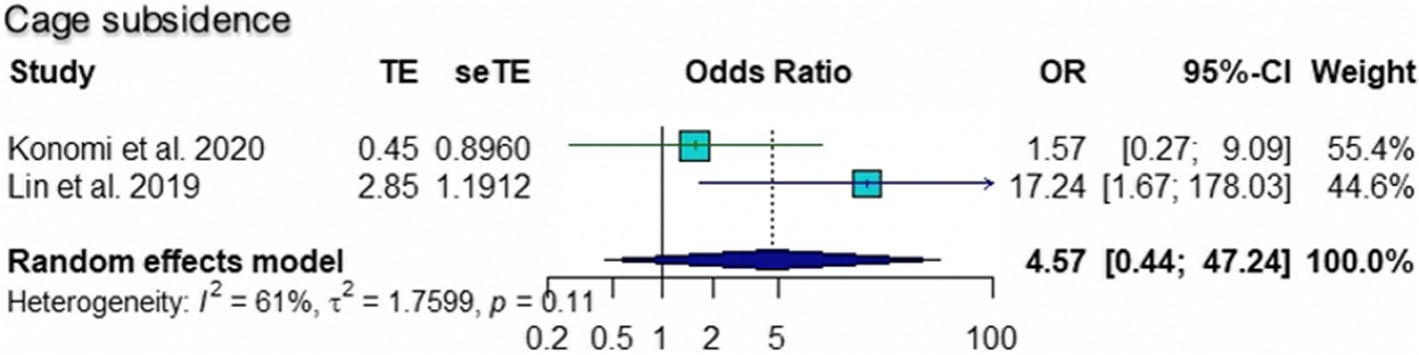
Forest plot and funnel plot of pooled odds ratio for cage subsidence

The pooled ORs for age (95% CI 1.02 to 1.11; *p* = 0.005), number of level fusions (95% CI 1.17 to 1.55; *p* < 0.0001), and smoking (95% CI 1.68 to 5.44; *p* = 0.0002) were found to be statistically significant risk factors for pseudarthrosis. The pooled ORs of other factors, including female sex (95% CI 0.31 to 5.19; *p* = 0.75), BMI (95% CI 0.81 to 1.21; *p* = 0.90), and cage subsidence (95% CI 0.44 to 47.24; *p* = 0.20), were not significantly different. According to the sensitivity analysis, the pooled OR for the number of level fusions was not significantly different when Hollern et al. [10] was omitted (95% CI = 0.78 to 2.10; *p* = 0.34). The results of common tests for publication bias showed evidence of bias according to age (Begg’s test, *p* = 0.60; Egger’s test, *p* = 0.10), number of level fusions (Begg’s test, *p* = 0.12; Egger’s test, *p* = 0.007), and smoking status (Begg’s test, *p* = 0.33; Egger’s test, *p* = 0.12). The trim and fill methods showed that there was no tremendous change in any factors. The forest plot and funnel plot of the pooled six risk factors, including the sensitivity analysis, are shown in Figs. 2, 3, 4, 5, 6 and 7.

### Subgroup analysis

We divided the included studies according to smoking status according to the duration of follow-up. The studies that mentioned smoking could be divided into two groups (12 months and 24 months) according to the duration of follow-up. For the 12-month group, there was no significant difference in the risk factor for pseudarthrosis, with a pooled OR of 1.89 (95% CI 0.88 to 4.16). For the 24-month group, smoking was a statistically significant risk factor for pseudarthrosis, with a pooled OR of 5.43 (95% CI 2.24 to 13.13). A forest plot of the pooled ORs from a subgroup analysis of the duration of follow-up is shown in Fig. 5.

## Discussion

Identifying the risk factors for pseudarthrosis is important for identifying preventive measures to decrease the incidence of this complication. Previous studies have reported a variety of risk factors. The present study included 12 publications for qualitative study and meta-analysis. There were 6 clinical risk factors, 5 radiographic risk factors, 4 surgical risk factors, and 2 bone turnover marker risk factors. The odds ratios (ORs) of the 6 risk factors mentioned in at least two publications were pooled. Age, number of level fuses, and smoking were reported to be risk factors for pseudarthrosis after lumbar fusion surgery.

The sensitivity analysis revealed that the pooled estimate of the effect of the number of level fuses was not significantly different when Hollern et al. [10] was omitted, while the pooled estimate of the effect of age and smoking did not change when the study was omitted. There was significant publication bias for age, number of level fusions, and smoking status, but the trim and fill method showed no significant changes. Subgroup analysis of the 12 months of follow-up revealed that the pooled ORs of smoking had no statistical significance for the risk of pseudarthrosis, which indicated that the duration of follow-up affected the smoking status.

How et al. [22] performed a systematic review and meta-analysis of the risk factors for pseudarthrosis in spinal deformity patients and revealed that the risk factors for pseudarthrosis were age > 55 years, number of level fusions > 12 segments, smoking, thoracolumbar kyphosis ^>20°^, and fusion to the sacrum. Graft material, preoperative coronal alignment, postoperative analgesia, and sex had no impact on the fusion rate. Age, smoking status, and number of level fuses were reported to be the same risk factors.

Formica et al. [23] studied influencing factors related to the fusion rate in lumbar interbody fusion surgery, which included age, osteoporosis, DM, obesity, and smoking. Age and smoking status were the same risk factors for pseudarthrosis in patients who underwent interbody fusion procedures reported in this study.

A past report distinguished risk factors, including age, DM status, BMI, and cage subsidence, as significant risk factors for the development of pseudarthrosis after lumbar fusion. Age was the main variable announced in our study.

Age is a typical risk factor that has been distinguished in many reviews. The inquiry is “How old is enough?”. How et al. referenced that an age more than 55 was critical. Be that as it may, Konomi et al. detailed factual importance at ages more established than 75 years. As of now, the cut-off is dubious.

Smoking was the most well-known clinical risk factor. A systematic review and meta-analysis by Nunna et al. [5] revealed that smoking was related to an expanded risk of pseudarthrosis contrasted with not smoking ≥ 1 year following spine surgery (RR 1.91, 95% CI 1.56 to 2.35). The outcome was not changed whether 1-level or 2-level fusion, allograft, or autograft was utilized. Glassman et al. [6] detailed that the frequency of pseudarthrosis was not essentially impacted by either the amount that a patient smoked before surgery or the duration of preoperative smoking cessation. Conversely, postoperative smoking cessation for more than 6 months was related to a diminished risk of pseudarthrosis. Likewise, in the present study, smoking was a risk factor for pseudarthrosis at two years of follow-up. In this way, smoking discontinuance ought to be encouraged for each smoker going through lumbar fusion to diminish the frequency of pseudarthrosis.

The number of levels fused was the only surgical risk factor for pseudarthrosis in the present study. However, the cut-off for how many levels affect the outcome is still unclear. Holfer et al. [24] reported that fusion at 4–8 levels and fusion at more than 9 levels were risk factors for pseudarthrosis. Jung et al. [17] also reported that fusion of more than 3 levels was a risk factor for pseudarthrosis, and How et al. [22] reported that fusion of more than 12 levels was a risk factor for pseudarthrosis. However, long fusion constructions should be performed cautiously.

This study has several limitations. First, we had a limited number of patients (1,830 patients). Second, most of the studies were retrospective (9 of 12). Third, 83.33% (10 of 12) of the included studies had a NOS of 5–7, which indicates moderate quality. Finally, we reviewed only English publications. In the future, a more prospective cohort study is needed to prove the effectiveness of these independent risk factors.

## Conclusion

The independent risk factors for pseudarthrosis in patients undergoing lumbar fusion procedures can be categorized into clinical risk factors, radiographic risk factors, surgical risk factors, and bone turnover marker risk factors. The meta-analysis demonstrated that age and smoking status were significant risk factors. The number of level fuses is a significant surgical risk factor. A more prospective study is needed to further investigate the other risk factors and the effect of surgery type and graft type on each factor.

## Data Availability

The datasets during and/or analysed during the current study available from the corresponding author on reasonable request.

## References

[1] Tsutsumimoto T, Shimogata M, Yoshimura Y, Misawa H (2008). Union versus nonunion after posterolateral lumbar fusion: a comparison of long-term surgical outcomes in patients with degenerative lumbar spondylolisthesis. EurSpine J.

[2] Sakai Y, Kaito T, Takenaka S, Yamashita T, Makino T, Hosogane N (2019). Complications after spinal fixation surgery for osteoporotic vertebral collapse with neurological deficits: Japan Association of Spine Surgeons with ambition multicenter study. J Orthop Sci.

[3] Derman PB, Singh K (2020). Surgical strategies for the treatment of lumbar pseudarthrosis in degenerative spine surgery: a literature review and case study. HSS J.

[4] Raizman NM, O’Brien JR, Poehling-Monaghan KL, Yu WD (2009). Pseudarthrosis of the spine. J Am Acad Orthop Surg.

[5] Nunna RS, Ostrov PB, Ansari D, Dettori JR, Godolias P, Elias E, et al. The Risk of Nonunion in Smokers Revisited: A Systematic Review and Meta-Analysis. Global Spine J. 2022;12(3):526–39. 10.1177/21925682211046899 PMC9121161 34583570

[6] Glassman SD, Anagnost SC, Parker A, Burke D, Johnson JR, Dimar JR (2000). The effect of cigarette smoking and smoking cessation on spinal fusion. Spine (Phila Pa 1976).

[7] Lee CS, Chung SS, Choi SW, Yu JW, Sohn MS (2010). Critical length of fusion requiring additional fixation to prevent nonunion of the lumbosacral junction. Spine (Phila Pa 1976).

[8] Gologorsky Y, Skovrlj B, Steinberger J, Moore M, Arginteanu M, Moore F (2014). Increased incidence of pseudarthrosis after unilateral instrumented transforaminal lumbar interbody fusion in patients with lumbar spondylosis: Clinical article. J Neurosurg Spine.

[9] GA Wells BS, D O’Connell, J Peterson, V Welch, M Losos, P Tugwell. The Newcastle‒Ottawa Scale (NOS) for assessing the quality of nonrandomized studies in meta-analyses. Available from: https://www.ohri.ca//programs/clinical_epidemiology/oxford.Asp.

[10] Hollern DA, Woods BI, Shah NV, Schroeder GD, Kepler CK, Kurd MF (2019). Risk factors for pseudarthrosis after surgical site infection of the spine. Int J Spine Surg.

[11] Suda K, Ito M, Abumi K, Haba H, Taneichi H, Kaneda K (2006). Radiological risk factors for pseudoarthrosis and/or instrument breakage after PLF with the pedicle screw system in isthmic spondylolisthesis. J Spinal Disord Tech.

[12] Otsuki B, Fujibayashi S, Tanida S, Shimizu T, Murata K, Matsuda S (2021). Possible association of pedicle screw diameter on pseudoarthrosis rate aftertransforaminal lumbar interbody fusion. World Neurosurg.

[13] Bydon M, De la Garza-Ramos R, Abt NB, Gokaslan ZL, Wolinsky JP, Sciubba DM (2014). Impact of smoking on complication and pseudarthrosis rates after single- and 2-level posterolateral fusion of the lumbar spine. Spine (Phila Pa 1976).

[14] Bydon M, De La Garza-Ramos R, Abt NB, Macki M, Sciubba DM, Wolinsky JP (2015). Durotomy is associated with pseudoarthrosis following lumbar fusion. J Clin Neurosci.

[15] Fujibayashi S, Takemoto M, Izeki M, Takahashi Y, Nakayama T, Neo M (2012). Does the formation of vertebral endplate cysts predict nonunion after lumbar interbody fusion?. Spine (Phila Pa 1976).

[16] Inose H, Yamada T, Mulati M, Hirai T, Ushio S, Yoshii T (2018). Bone turnover markers as a new predicting factor for nonunion after spinal fusion surgery. Spine (Phila Pa 1976).

[17] Jung JM, Chung CK, Kim CH, Yang SH, Ko YS. Prognosis of Symptomatic Pseudarthrosis Observed at 1 Year After Lateral Lumbar Interbody Fusion. Spine (Phila Pa 1976). 2021;46(18):E1006–13. 10.1097/BRS.0000000000003980 33534522

[18] Konomi T, Yasuda A, Fujiyoshi K, Yato Y, Asazuma T (2020). Incidences and risk factors for postoperative non-union after posterior lumbar interbody fusion with closed-box titanium spacers. Asian Spine J.

[19] Satake K, Kanemura T, Nakashima H, Ishikawa Y, Segi N, Ouchida J (2018). Nonunion of transpsoas lateral lumbar interbody fusion using an allograft: clinical assessment and risk factors. Spine Sur Rel Res.

[20] Macki M, Syeda S, Rajjoub KR, Kerezoudis P, Bydon A, Wolinsky JP (2017). The effect of smoking status on successful arthrodesis after lumbar instrumentation supplemented with rhBMP-2. World Neurosurg.

[21] Lin GX, Kotheeranurak V, Zeng TH, Mahatthanatrakul A, Kim JS (2019). A longitudinal investigation of the endplate cystic lesion effect on oblique lumbar interbody fusion. Clin Neurol Neurosurg.

[22] How NE, Street JT, Dvorak MF, Fisher CG, Kwon BK, Paquette S (2019). Pseudarthrosis in adult and pediatric spinal deformity surgery: a systematic review of the literature and meta-analysis of incidence, characteristics, and risk factors. Neurosurg Rev.

[23] Formica M, Vallerga D, Zanirato A, Cavagnaro L, Basso M, Divano S (2020). Fusion rate and influence of surgery-related factors in lumbar interbody arthrodesis for degenerative spine diseases: a meta-analysis and systematic review. Musculoskelet Surg.

[24] Hofler RC, Swong K, Martin B, Wemhoff M, Jones GA (2018). Risk of pseudoarthrosis after spinal fusion: analysis from the healthcare cost and utilization project. World Neurosurg.

